# Unexpected Return for Follow-up During the First Year of Multidisciplinary Care May Be Predictive of Rapid Deterioration of Renal Function

**DOI:** 10.1097/MD.0000000000001731

**Published:** 2015-10-16

**Authors:** Ming-Hsien Tsai, Yu-Weil Fang, Li Hui Wang, Xiang Gin You, Jyh-Gang Leu

**Affiliations:** From the Division of Nephrology, Department of Internal Medicine, Shin-Kong Wu Ho-Su Memorial Hospital (M-HT, Y-WF, LHW, XGY, J-GL); Fu-Jen Catholic University School of Medicine (J-GL); and Division of Biostatistics, Institutes of Epidemiology and Preventive Medicine, College of Public Health, National Taiwan University, Taipei, Taiwan (M-HT).

## Abstract

Multidisciplinary predialysis education and team care (MDC) may slow the decline in renal function in patients with chronic kidney disease (CKD). However, associations between unexpected return during MDC and progression of renal dysfunction have not been characterized in patients with CKD. Our study aimed to determine the association between exacerbation of renal dysfunction and the frequency of unexpected return during follow-up.

A total of 437 patients with CKD receiving multidisciplinary care between January 2009 and June 2013 at the Shin-Kong Wu Ho-Su Memorial Hospital were included in this retrospective observational cohort study, and multiple imputations were performed for missing data. The predictor was the frequency of unexpected return for follow-up during the first year after entering MDC. Main outcome was monthly declines in estimated glomerular filtration rates (eGFR). Moreover, the demographic data, comorbidities, history of medication, and routine laboratory data for patients with CKD were collected.

Among all patients, 59.7% were male, the mean age at initiation of MDC was 69.4 ± 13.2 years, and the duration of follow-up was 21.4 ± 3.3 months. The subjects were divided into 2 groups according to frequencies of follow-up (≤4 and > 4 visits) during the 1st year of MDC. The patients with CKD were regularly followed up every 3 months as a part of MDC in our hospital, and patients who returned for more than 4 follow-up visits were included in the unexpected return group. In crude regression analyses, unexpected return was significantly associated with higher monthly declines of eGFR (β = 0.092, 95% confidence interval, 0.014–0.170). This association remained after adjustments for multiple variables, and subgroup analyses of unexpected return showed that male gender, older age, CKD stage 1 to 3, hypertension, history of coronary artery disease, and use of renin–angiotensin system blockade were significantly associated with declines in renal function.

In conclusion, unexpected return for follow-up during the 1st year of MDC was significantly associated with the deterioration of renal function.

## INTRODUCTION

Chronic kidney disease (CKD) is recognized as a global public health problem,^1–3^ and care for patients with CKD is complicated because of high risks of morbidity and mortality. Consequently, some patients with CKD under the care of primary physicians or other specialists may not receive optimal care. In particular, late referral to nephrologists is associated with poor outcomes and increased mortality and morbidity,^4–6^ whereas early referral to nephrology departments allows sufficient predialysis education, which can delay the initiation of dialysis and improve mortality rates.^7,8^ However, predialysis nephrology care fails to decrease the social impact of CKD,^9^ and a cooperative intervention with nephrologist-based multidisciplinary care (MDC) was developed to improve positive attitudes for disease management among patients with CKD. This level of care has a substantial influence on mortality and morbidity and delays entry into hemodialysis.^10–12^

Although the national prevalence of CKD is high in Taiwan, the awareness is inadequate and only 3.5% of patients can report their stage of disease.^3^ Accordingly, a unique protocol to standardize and regulate pre-end-stage renal disease care has been established as a part of the medical system in Taiwan, and all medical costs are covered by the National Health Insurance. Specifically, Chen et al^10^ showed improved survival rates, control of mineral bone disease, and slower declines of renal function in patients with CKD receiving MDC in Taiwan. However, patients with CKD do not benefit equally from MDC programs and individualized MDC programs may be required. Therefore, identification of risk factors for declines in renal function after entry into MDC programs is warranted. Hence, because some patients with CKD returned to the MDC program with unexpectedly high frequency, we investigated associations between unexpected return to MDC programs and declines in renal function.

## METHODS

### Study Population

**FIGURE 1:**
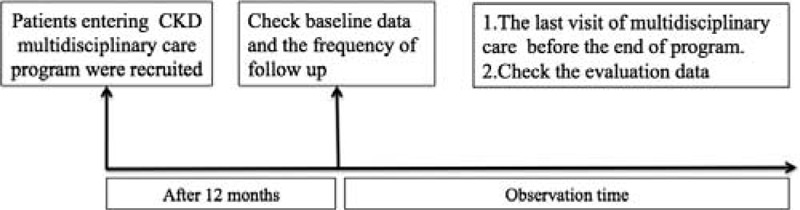
Flowchart of the study design.

This retrospective cohort study was conducted at a single medical center and included patients who had entered the MDC program for CKD from outpatient nephrology clinics between January, 2009 and June, 2013 at Shin-Kong Wu Ho-Su Memorial Hospital, Taipei, Taiwan. Patients who had participated for less than 12 months, had any malignancy, or had been included in another study were excluded from the analyses. A total of 437 patients were observed until June 2013, as shown in the study scheme (Figure 1). The Institutional Review Board of the Shin-Kong Wu Ho-Su Memorial Hospital, Taipei, Taiwan approved the study and waived the requirement of informed consent, because the study was based on medical chart reviews. All patient information was anonymized and deidentified prior to analysis.

### Multidisciplinary Care (MDC)

MDC was provided by a nephrologist, a nephrology nurse educator, a renal dietitian, a social worker, a pharmacy specialist, and a surgeon, who performed vascular access placements and peritoneal dialysis tube (tenchoff catheter) implantations.^10^ Standardized interventions for CKD in the MDC program included management and education according to CKD stages and were performed with reference to the National Kidney Foundation/Kidney Disease Outcomes Quality Initiative guidelines^13^ and the Taiwan predialysis care program. CKD management in the MDC group was focused on medical management and lifestyle modification. Stage III or IV patients with CKD were followed up every 3 months, and stage V patients with CKD were followed up at least every month. Declines in renal function were associated with progression of uremia symptoms in pre-ESRD, and patients with CKD increased the frequency of their visits to every 2 weeks or a week. However, poor patient compliance and the reimbursement policy of the National Health Insurance in Taiwan compromised the follow-up of visiting frequencies. Thus, we invited patients of all CKD stages to return for scheduled education and evaluation every 3 months after joining MDC program in our hospital. The case-management nephrology nurse contacted patients to ensure regular follow-ups, and an unexpected return was defined as greater than 4 visits per year.

### Data Collection

All patients completed a structured questionnaire and socio-demographic characteristics, and lifestyles were recorded upon entry into the MDC program. Demographic information was collected, and anthropometric indices; blood pressure; biochemical measurements; histories of comorbidity; and medications, including renin–angiotensin system (RAS) and lipid lowering agents, were recorded. Baseline clinical and laboratory data were collected after participation in the MDC for a year, and blood pressure and levels of blood nitrogen, creatinine (Cr), sodium (Na), potassium (K), ionized calcium (iCa), phosphate (P), albumin, uric acid, total cholesterol (TC), triglyceride (TG), hematocrit (Hct), and HbA1c, and urine protein–creatinine ratios (UPCRs) were determined at the last visit before the end of study. Estimated glomerular filtration rates (eGFRs) were estimated using the modification of diet in renal disease equation,^14^ and mean monthly declines in eGFR ([baseline eGFR-last eGFR]/time period) were analyzed as the primary outcome.

### Statistical Analyses

Multiple imputations were used to accommodate missing data and the Markov chain Monte Carlo method^15^ yielded unbiased results with accurate estimates of standard errors from the present data under the assumption that the data are multivariate normally distributed.^16,17^ Five complete datasets were generated and combined for analytical inference. Data are expressed as the mean ± standard deviation and median (25th, 75th centile) or frequency, as appropriate. Patients with an unexpected return for follow-up were grouped and compared with normal patients using the χ^2^ test for categorical variables and the independent *t*-test for continuous variables. Subsequently, multiple logistic regression analysis was used to identify the risk factors associated with an unexpected return episode in MDC (frequency of follow-up >4). A modified stepwise procedure with 6 modeling steps was performed to investigate the independent associations between the frequency of follow-up (>4 vs ≤4 visits) and monthly declines in eGFR. The 1st model was crude analysis. The 2nd model consisted of age, sex, CKD stage, and follow-up duration. The 3rd model consisted of adding comorbidity and medication history. The 4th and 5th steps were adding biochemical factors. The final step was entering nutritional markers into the model. Moreover, we performed subgroup analysis by the factors of gender, age, CKD stage, diabetes mellitus (DM), coronary artery disease (CAD), hypertension, and RAS blockade. A *P*-value of ≤0.05 was considered statistically significant. All statistical analyses were performed using SAS 9.3 (SAS institute, Inc., Cary, NC) and Statistical Package for the Social Sciences (SPSS) 16.0 (SPSS, Chicago, IL) softwares.

## RESULTS

**FIGURE 2:**
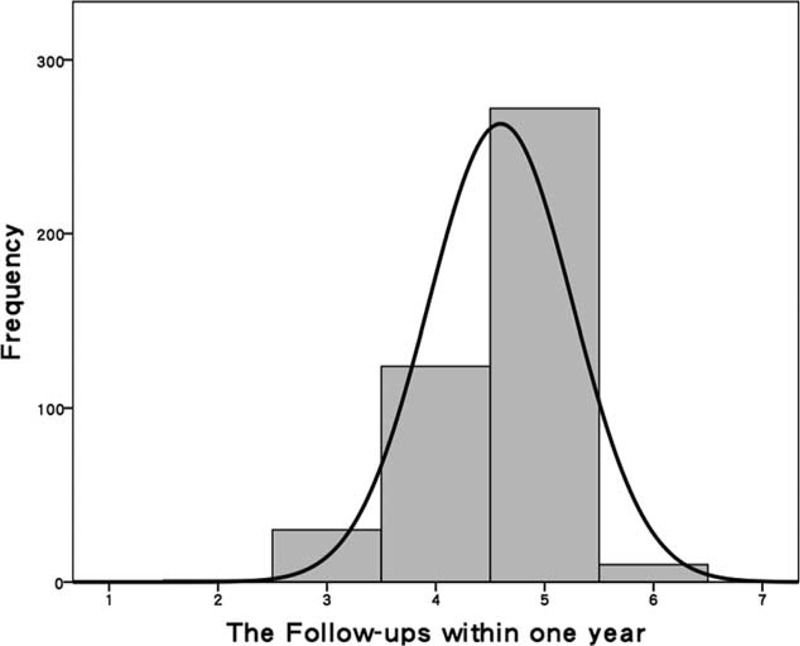
Frequency of follow-up during the 1st year of participation in the multidisciplinary predialysis education and team care program.

The mean age of participants was 69.4 ± 13.2 years, and the mean follow-up period was 21.4 ± 3.3 months. During the 1st year of participation in the MDC care program (Figure 2), 155 patients (35.4%) had ≤4 follow-up visits and 282 participants (64.6%) had >4 follow-up visits. Missing data included determinations of serum uric acid (3.9% missing), serum Na (4.6%), serum K (1.8%), serum iCa (9.2%), serum P (7.6%), serum TC (27.2%), serum TG (3.9%), HbA1c (7.1%), and UPCR (11%) (Table 1). Patient groups were equivalent in age, gender, CKD stage, length of follow-up, comorbidity of DM, hypertension, gout and CAD, and biologic values at baseline (Table 2). However, RAS blockade usage differed significantly between the groups.

**TABLE 1:**
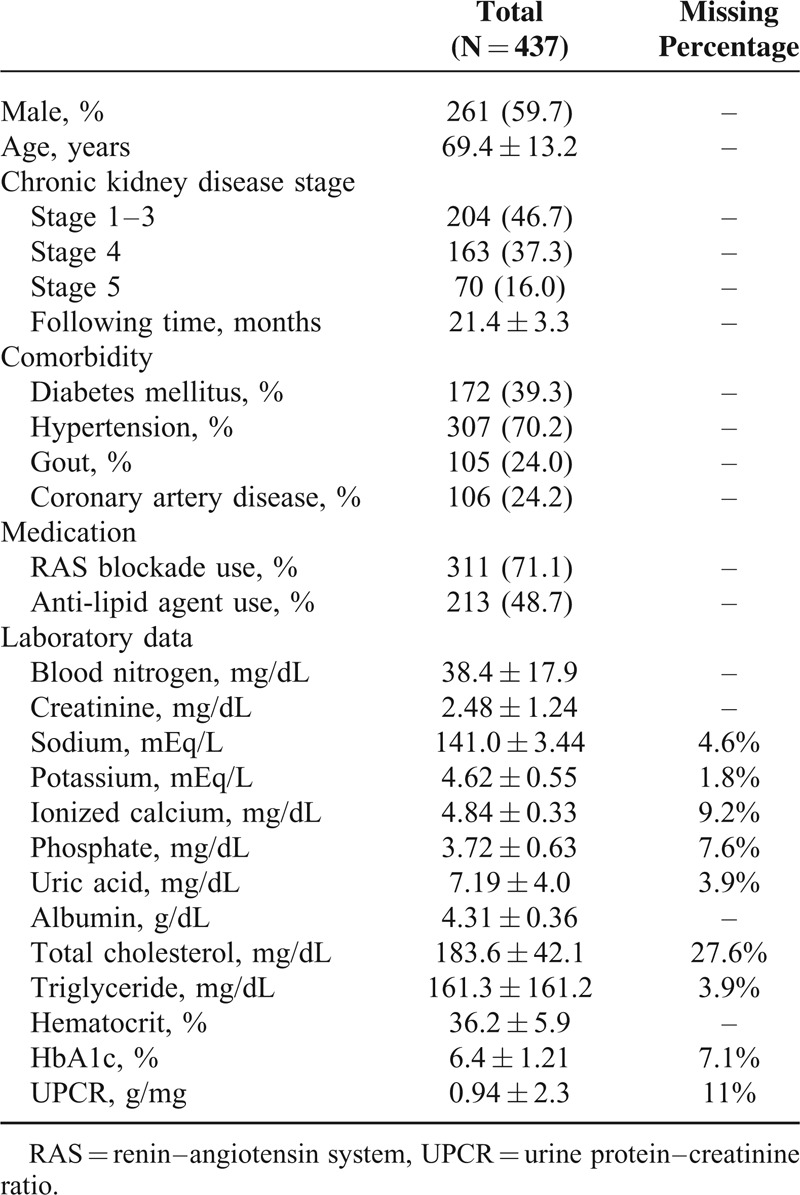
Characteristics of Study Population

**TABLE 2:**
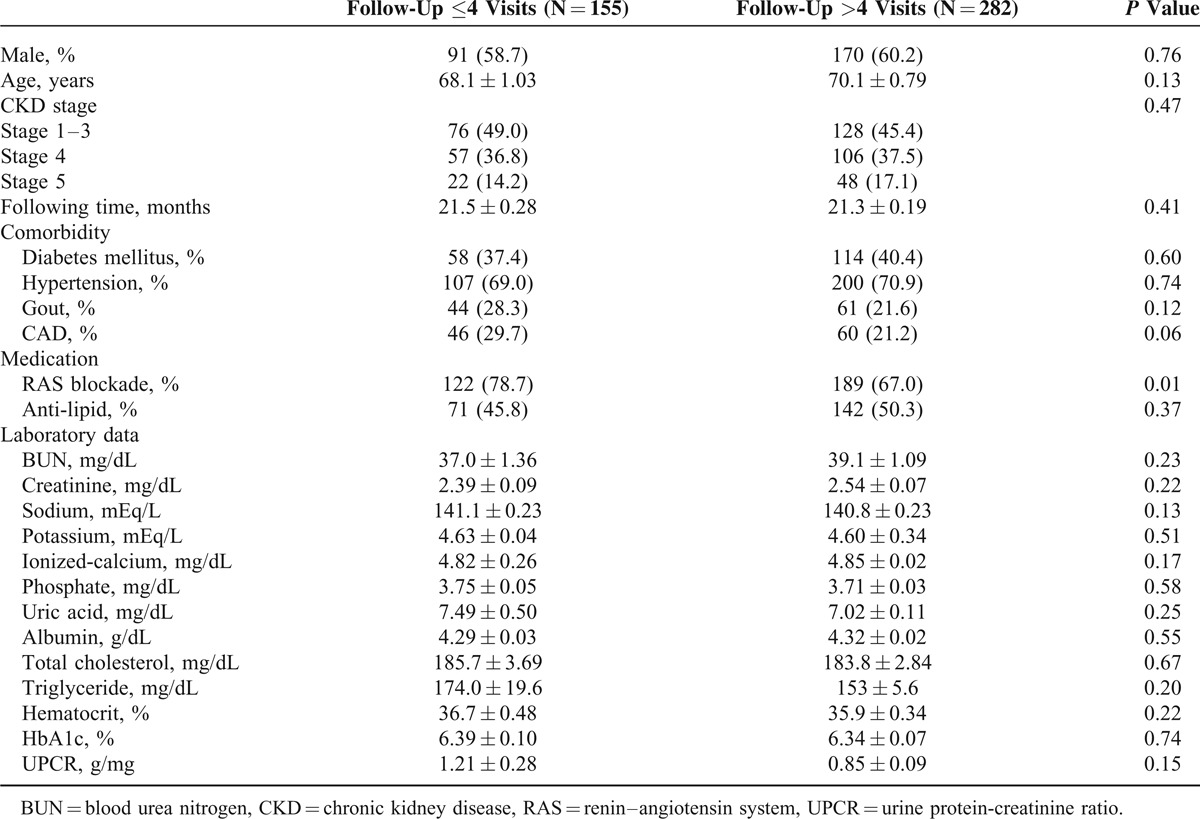
Characteristics of the Study Population Stratified by Follow-Up Frequency

### Risk Factors for Unexpected Return of Follow-Up

Determinants of frequency of follow-up were identified in univariate regression analysis (Table 3). Unexpected return was significantly associated with RAS blockade usage (odds ratio [OR], 0.55; 95% confidence interval [CI], 0.34–0.89) and was insignificantly associated with CAD (OR, 0.64; 95% CI, 0.40, 1.00). Subsequent multivariate analysis identified CAD (adjusted OR, 0.49; 95% CI, 0.29, 0.82) as a significant determinate of unexpected return for follow-up. Moreover, the predictability of RAS blockade usage was reduced but near significant (adjusted OR, 0.65; 95% CI, 0.39–1.08) after adjustment for gender, age, CKD stage, observation time, DM, hypertension, CAD, antilipid agent use and the levels of albumin, Hct, and UPCR.

**TABLE 3:**
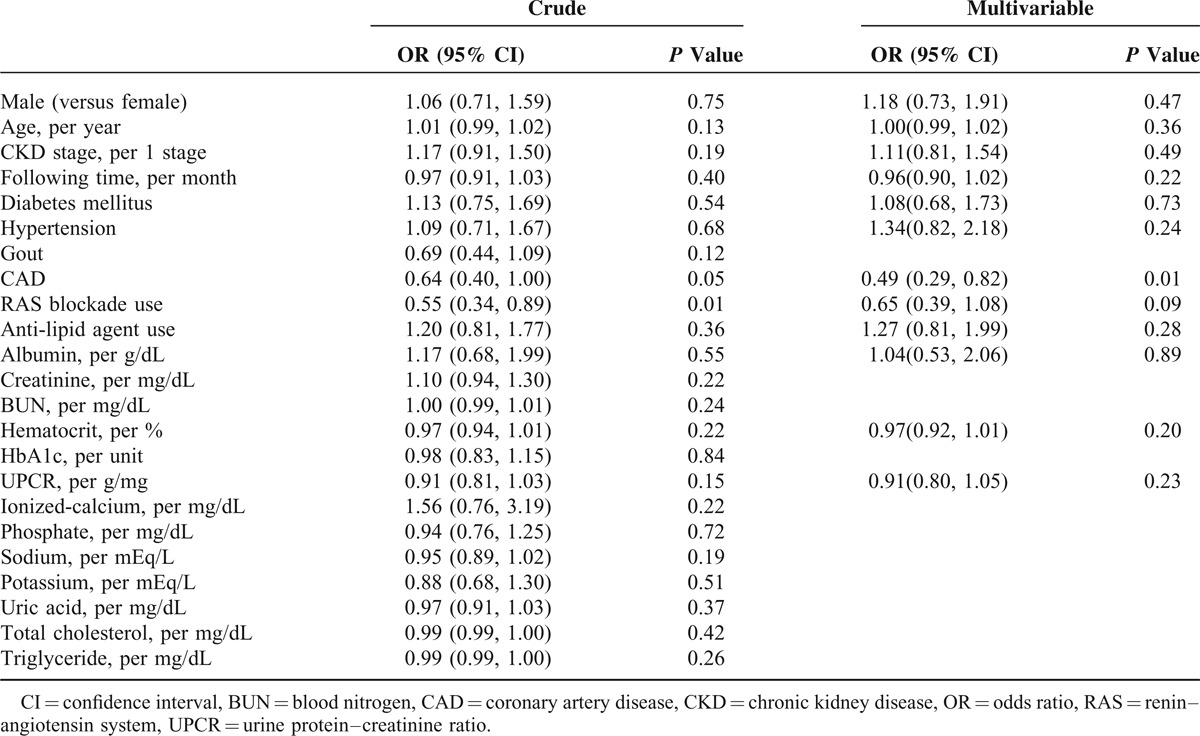
Determinants of >4 Follow-Up Visits

### eGFR Decline in Unexpected Return

Decline in eGFR was higher in the unexpected return group and the associated was significant in unadjusted analyses (β = 0.092, 95% CI, 0.014–0.170) and in multivariate models that were adjusted for age, gender, CKD stage, and follow-up time (β = 0.101, 95% CI, 0.023–0.179; Table 4). This relationship remained significant after further adjustment for DM, hypertension, CAD, RAS blockade, and use of antilipid medicine (β = 0.101, 95% CI, 0.023–0.179). Further adjustments for HBA1c, Hct, iCa, and P in regression model 4 (β = 0.092, 95% CI, 0.001–0.183) and for UPCR in regression model 5 (β = 0.096, 95% CI, 0.001–0.192) attenuated the association between monthly eGFR decline and unexpected return, but it remained significant. However, after adjustment for raw albumin, TG and TC values, this association became insignificant (β = 0.050, 95% CI, −0.040–0.139). In contrast, unexpected return was significantly associated with monthly eGFR declines in all regression analyses of imputed data.

**TABLE 4:**
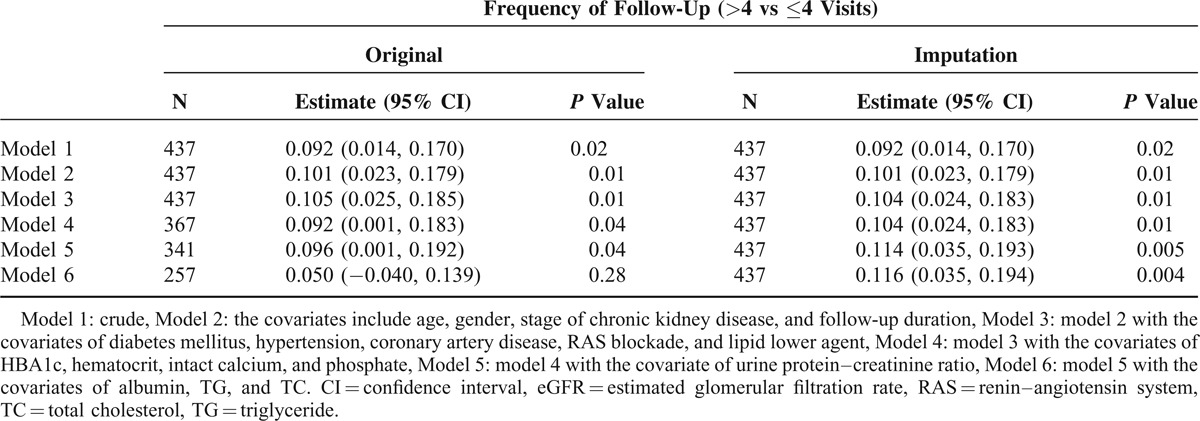
Multivariate Regression Analysis of Risk Factors for Monthly Declines in eGFR. Frequency of Follow-Up Was Recorded as a Dichotomous Variable

### Subgroup Analysis

Associations between unexpected return for follow-up and MDC and eGFR declines were investigated using covariates of gender, age (≤65 and >65 years), CKD stage (<4 and ≥4), DM, CAD, hypertension, and RAS blockade (Table 5). After multivariate adjustment for demographic characteristics, laboratory data, and follow-up duration, frequencies of >4 follow-up visits were significantly associated with eGFR declines in older patients, male patients, those with CKD stage < 4, hypertension, and no CAD or RAS blockade mediations. However, analyses of imputed data indicated a significant relationship with eGFR declines in older patients, male patients, those with CKD stage < 4, hypertension, RAS blockade use, and no CAD.

**TABLE 5:**
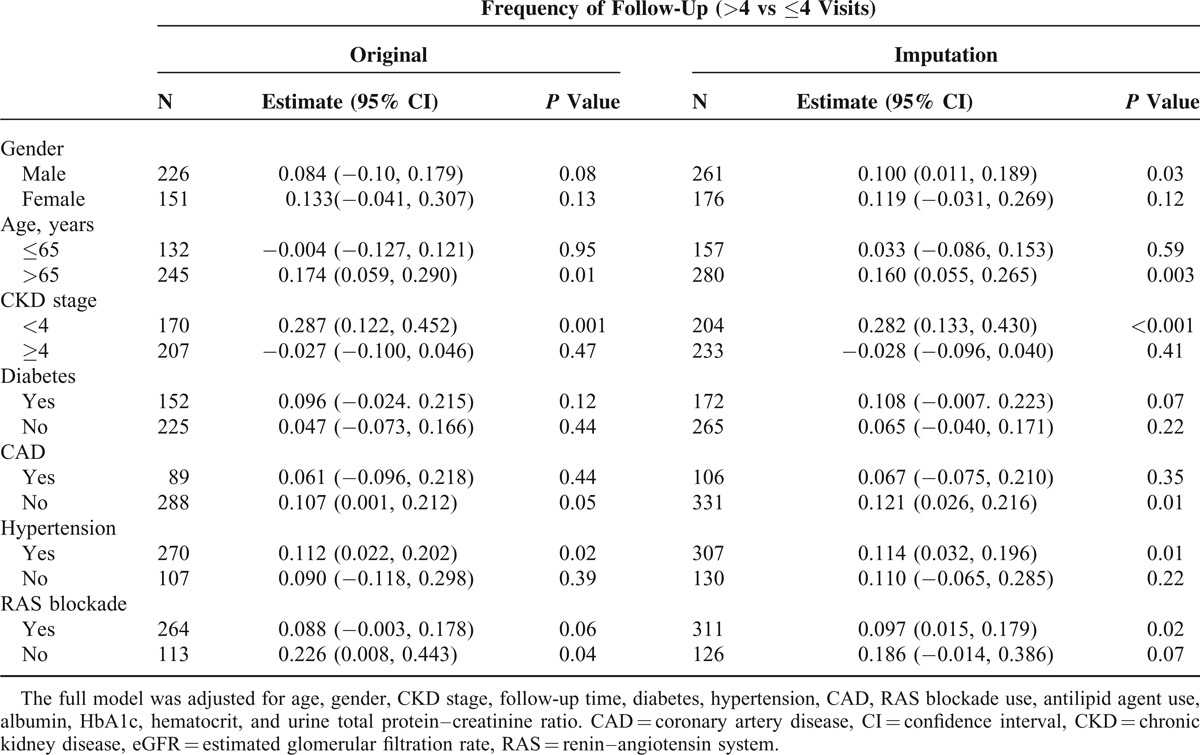
Subgroup Analysis of Monthly Declines in eGFR

## DISCUSSION

This retrospective cohort study demonstrated that episodes of unexpected return in the 1st year of MDC are significantly associated with a decline in renal function. In crude and multivariate analysis, patients without RAS blockade or CAD had a higher risk of unexpected return during the 1st year of MDC. Although frequent education meetings of MDC have been shown to preserve renal function in previous studies, the present data show no benefits among patients who returned for more than 4 follow-up visits.

Etiological interpretations of the present data were not possible because the reasons for unexpected return during participation in the MDC program were not assessed. However, patients without histories of CAD or RAS blockade use had a propensity for unexpected return. Numerous studies have demonstrated the protective effects of these agents on renal function in patients with CKD, mainly in terms of diminished proteinuria,^18–20^ and CKD is a known risk factor for cardiovascular diseases (CVD).^21,22^ Thus, unexpected return among patients without CAD or RAS blockade use may reflect motivation by more advanced renal exacerbation and the onset of CVD events. Accordingly, the present subgroup analyses showed a stronger relationship between unexpected return and renal exacerbation in older (>65-years old) male patients and in those with mild CKD, DM, hypertension, RAS blockade use, and without CAD. These observations warrant careful evaluations of patient clinical conditions upon unexpected return for follow-up.

Adjustments of the present stepwise linear regression models for albumin, TC, and TG weakened the association between renal exacerbation and unexpected return, potentially reflecting the frequency of missing TC data (27.6%). However, unexpected return remained a significant predictor of renal deterioration after multiple imputations for missing values, indicating that unexpected return during the 1st year of participation in the MDC is an independent risk factor for reduced renal function regardless of demographic characteristics, CVD, DM, nutrition status, or proteinuria.

CKD is increasingly prevalent in developed countries such as the United States,^23,24^ and the effects of progressive CKD significantly compromise quality of life and consume healthcare resources. Previous reports suggest that patients with CKD benefit from comprehensive MDC and that an integrated healthcare system may improve health outcomes.^10–12^ However, these benefits vary among patients with diverse disease conditions. Hence, the uniform educational component of MDC may not be appropriate for all patients warranting careful identification of high risk patients in MDC. Accordingly, the present data suggest that patients who returned unexpectedly for follow-up during the 1st year had poor renal outcomes than others in the CKD MDC, further indicating the need for individualized educational components of MDC for this group.

The present data are limited to a single center, which may not be representative of all CKD populations. The further study with large-scale and multicenter design is needed to verify our finding. Moreover, only baseline covariates were used to predict declines of renal function, potentially introducing biased estimates of temporal predictors. The reasons of unexpected return were not evaluated, which may induce the bias of confounding by indication. However, our purpose is to suggest further attention in the CKD patients with setting. Finally, although some important parameters of renal function decline were not assessed in some patients, the present multiple imputations have been validated for data reconstruction. To our knowledge, this is the 1st study that demonstrated an association between unexpected return for follow-up during the 1st year of participation in the MDC program and renal function declines in patients with CKD, thus indicating the need for increased attention for these patients at MDC centers.

## CONCLUSION

MDC offers a holistic approach to treatments for patients with CKD, and consequent improvements in renal function have been demonstrated. However, unexpected return for follow-up during the 1st year of MDC may indicate risks of rapid deterioration of renal function. The present data indicate the requirement of special attention for these patients and warrant further studies to identify subsets of MDC-participating patients with CKD who are at a risk of poor clinical outcomes and to develop a tailored program for them.
